# Evaluating cellular responses of lung and liver cells to graphene oxide functionalized poly(propylene)imine and polyamidoamine dendrimers: insights for biomedical applications

**DOI:** 10.1186/s11671-026-04636-0

**Published:** 2026-09-11

**Authors:** Beatriz Fumelli Monti Ribeiro, Julyane Batista Chaves, Harsh Jain, Siyu Fan, Guilherme Luiz Souza de Jesus, Devaney Ribeiro do Carmo, Romana Schirhagl, Gláucia Maria Machado-Santelli

**Affiliations:** 1 Department of Cell and Developmental Biology, University of São Paulo (USP), São Paulo, 05508900 Brazil; 2 Department of Biomaterials and Biotechnology, Groningen University, University Medical Center Groningen, Groningen, 9713AV The Netherlands; 3 Department of Physics and Chemistry, School of Engineering, São Paulo State University (UNESP), Ilha Solteira, SP 15385-007 Brazil

**Keywords:** Graphene oxide, Dendrimers, Cell viability and cell morphology

## Abstract

10 million people die of different forms of cancer every year. One of the largest problems is the great variability between patients in drug efficacy, that is partly due to the low specificity of cancer drugs and their consequent severe side effects, which make it difficult to avoid damage to healthy tissues. To address these challenges, several targeted therapies and drug delivery strategies have been developed that can selectively act on cancer cells to improve efficacy and reduce toxicity. One promising approach is the use of graphene or graphene oxide-based materials for this purpose. Here we investigated a graphene oxide (GOX) composite material functionalized with 2 different dendrimers: poly(propylene)imine (DAB-Am-16) and polyamidoamine (PAMAM). These dendrimers were covalently bound to GOX via amide formation between any peripheral amine group of the dendrimers and the carboxyl groups present in GOX. We explored the cellular responses of the materials on BEAS-2B (non-tumoral), LC-HK2 and HepG2 cancer cells. We investigated cell viability, morphological changes in the cytoskeleton organization, the spatial interaction between the materials and the cell nuclei. Our results showed that GOX materials reduced cell viability at higher concentrations, and cellular exposure to these materials was associated with cytoskeletal reorganization, including reduced cytoplasmic and nuclear areas. These findings highlight their potential as promising candidates for biomedical applications.

**Supplementary Information:**

The online version contains supplementary material available at https://doi.org/10.1186/s11671-026-04636-0.

## Introduction

Cancer is one of the biggest global health problems and many treatments fail in response due to the development of drug resistance and metastasis. Therefore, it is crucial to explore new treatment alternatives for the disease. For this reason, researchers have intensively studied tumor biology to define new therapeutic targets, as well as the action of new compounds and optimize their administration strategies, such as using graphene oxide (GOX) for the delivery of anti-cancer drugs [1, 2, 3].

GOX is a highly oxidized form of graphite [4]. It is a two-dimensional carbon sheet which has oxygen-containing functional groups, such as hydroxyl, carboxyl, and epoxy. These groups allow the association with different molecules [5, 6].

The functionalization of GOX with other materials, such as dendrimers, proves to be an interesting strategy for producing hybrid nanomaterials in different formulations [7, 8] and this association produces more biocompatible and versatile materials [9]. Besides traditional polymers, dendrimers have received considerable interest in biomedical applications due to their precise molecular weight, multifunctionality, biocompatibility, and excellent water solubility [10].

However, the increasing applicability of graphene-based materials in therapy and diagnosis requires a comprehensive assessment of their biocompatibility and it is still necessary to fully understand their risks and their toxic effects in different cell lines [11, 12].

The uptake of graphene materials by cells has been previously reported in many studies [13, 14, 15, 16]. GOX sheets exhibit affinity for the nuclear membrane, influencing cellular metabolism and proliferation [17]. Other authors showed that these materials are also capable of causing disorganization in the actin cytoskeleton and modifying cell migration [18]. Endocytosis is the mechanism reported for the cellular uptake of GOX dots [19] which are smaller than our material.

Additionally, physico-chemical properties such as size, charge and composition are among the most important aspects to comprehend, because they can influence the bioavailability and internalization process [20, 21, 22].

Therefore, the objective of this study was to evaluate the cellular responses induced by three graphene oxide-based materials: unmodified GOX, GOX functionalized with third-generation poly(propylene imine) dendrimer (GOXD), and GOX functionalized with poly(amidoamine) dendrimer (GOXP), in lung and liver cell models. Specifically, we investigated effects on cell viability, cytoskeletal organization, nuclear morphology, and nanoparticle-cell spatial interactions to better understand their biological behavior.

**Fig. 1:**
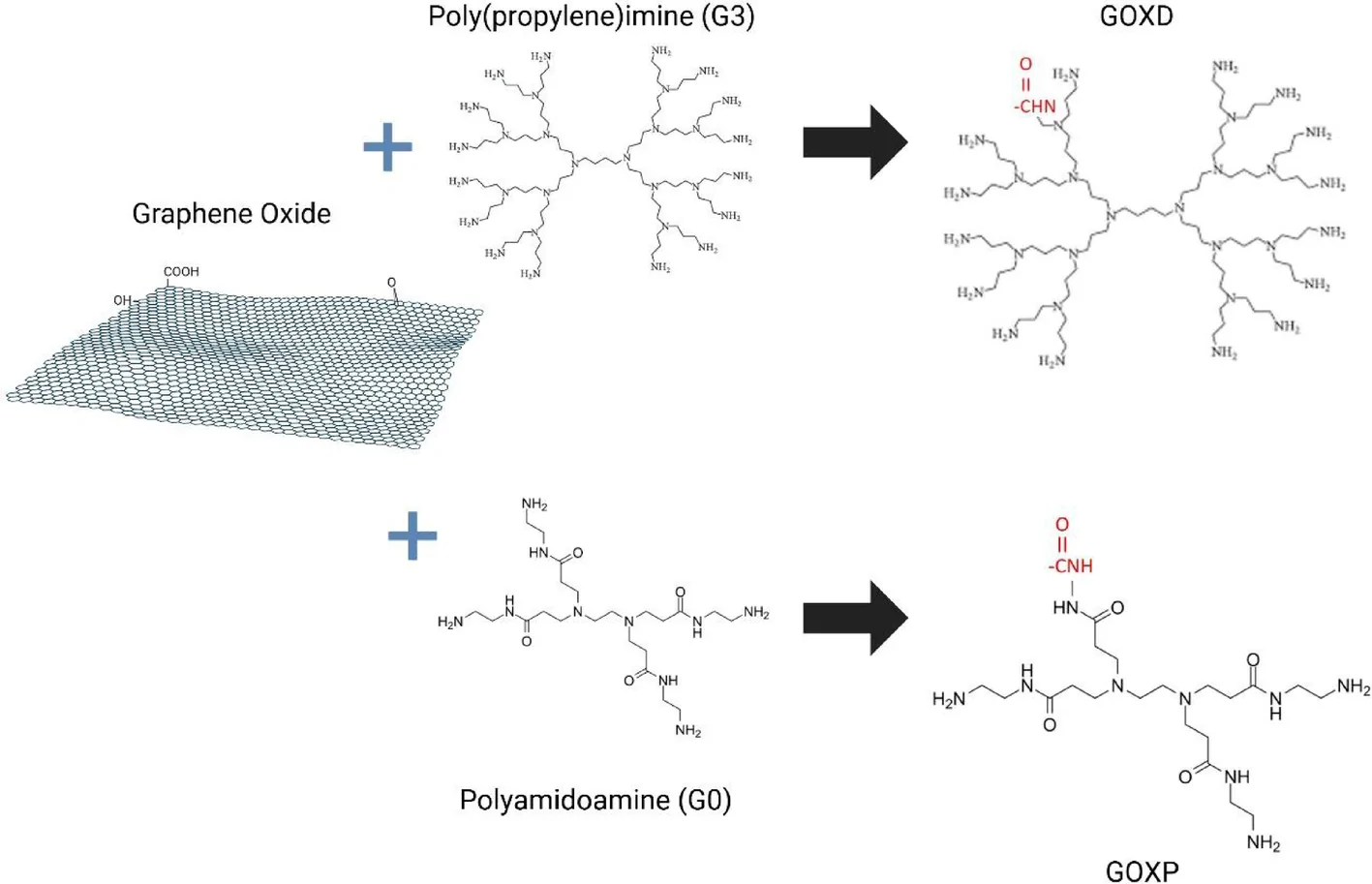
Synthesis process of the materials used in this work. Our starting material is Graphene oxide (GOX), to which we attached in B polyamidoamine dendrimer PAMAM G.0 and poly(propylene)imine 3 Generation dendrimer (DAB-Am-16), resulting in GOXD and GOXP

These dendrimers were covalently attached to GOX through amide bond formation between their peripheral amine groups and the carboxyl groups on GOX (Fig. 1). Dendrimers offer several advantages, including enhanced structural definition resulting in reduced heterogeneity, moderate multivalency, low intrinsic cytotoxicity, and ease of surface functionalization and analytical characterization. Such properties make them particularly suitable for drug loading applications, as their branched architecture and terminal functional groups enable efficient conjugation of bioactive compounds [23, 24, 25].

We tested the materials on cell lines relevant to toxicological assessment. Specifically, cellular responses were evaluated in the non-tumoral lung epithelial cell line BEAS-2B, the tumoral lung cell line LC-HK2, and the hepatocellular carcinoma cell line HepG2. BEAS-2B cells were included to model pulmonary epithelial responses, as the respiratory tract represents a primary interface for nanoparticle exposure. The LC-HK2 cell line enabled comparison between non-malignant and malignant pulmonary cells, allowing evaluation of potential differential susceptibility to graphene oxide-based materials. HepG2 cells were selected due to the central role of the liver in nanoparticle accumulation, metabolism, and systemic distribution, making hepatic models essential for assessing nanoparticle-cell interactions and structural cellular responses [26, 27, 28, 29, 30, 31].

## Materials and methods

### Reagents

Graphene oxide powder (15–20 sheets, 4–10% edge‑oxidized), poly(amidoamine) dendrimer (PAMAM, generation 0), poly(propylene)imine dendrimer (DAB‑Am‑16, generation 3.0), N, N′‑dicyclohexylcarbodiimide (DCC), graphite powder, isoniazid, L‑dopamine, and nujol oil were purchased from Sigma‑Aldrich (USA) and used as received without further purification.

### Surface functionalization of graphene oxide powder with a polyamidoamine dendrimer (PAMAM G0)

Graphene oxide (GOX) was functionalized with poly(amidoamine) (PAMAM) dendrimer generation 0 via covalent attachment through amide bond formation. In a typical procedure, 1.0 g of graphene oxide powder was placed in a 100 mL round‑bottom flask containing 40 mL of methanol. Subsequently, 1.3 mL of PAMAM dendrimer generation 0 (per gram of GO) and 0.100 g of N, N′‑dicyclohexylcarbodiimide (DCC) were added as the coupling agent.

The resulting suspension was ultrasonicated for 10 min to ensure homogeneous dispersion and then refluxed for 24 h under an inert nitrogen atmosphere, promoting the reaction between the carboxylic groups on the graphene oxide surface and the terminal amine groups of the dendrimer. After completion of the reaction, the solvent was removed by rotary evaporation. The solid product was vacuum‑filtered and washed exhaustively with methanol to remove unreacted dendrimer and residual coupling reagent. The final material, denoted as GOP (graphene oxide-PAMAM), was dried in a vacuum oven at 50 °C, yielding a stable solid (GOXP).

### Functionalization of graphene oxide powder with poly(propylene)imine hexadecaamine dendrimer (DAB-Am-16)

In a typical procedure, 1.0 g of graphene oxide powder was dispersed in 50 mL of ethanol, followed by the addition of 0.672 g of poly(propylene)imine dendrimer (DAB‑Am‑16, generation 3.0) and 0.100 g of N, N′‑dicyclohexylcarbodiimide (DCC) as coupling agent. The mixture was ultrasonicated for 10 min to ensure homogeneous dispersion and then refluxed for 24 h under continuous stirring. After completion of the reaction, the solvent was removed by evaporation, and the solid product was vacuum‑filtered through a 0.22 μm PVDF membrane. The material was exhaustively washed with ethanol to remove unreacted dendrimer and residual reagents and subsequently dried in a vacuum oven at 70 °C, yielding the dendrimer‑functionalized graphene oxide (GOXD).

### Characterization of the materials

The hydrodynamic diameter, polydispersity index (PdI), and zeta potential of GOX, GOXD, and GOXP were determined by dynamic light scattering (DLS) at 25 °C using a Zetasizer Nano ZS90 (Malvern Instruments Ltd., UK). The stock dispersions were subjected to ultrasonication in a water bath for 30 min at room temperature to promote deagglomeration. The suspensions were subsequently diluted in MilliQ water to a final concentration of 10 µg/mL prior to dynamic light scattering (DLS) analysis.

We also employed a Nanoparticle Tracking Analysis (NTA) technique. This technique, similar to dynamic light scattering (DLS), relies on analyzing the Brownian motion of nanoparticles by detecting the light they scatter. A laser beam illuminates particles suspended in liquid, and by following the trajectory of each particle frame by frame over time, the software determines their diffusion coefficients. This approach enables the accurate quantification of nanoparticle and size distribution [32, 33]. NTA measurements were performed in a ZetaView Particle Metrix PMX x30 (Particle Metrix, Germany). For the measurements, the samples were diluted to 0.2 µg/mL in MiliQ water and injected into the NTA chamber with sterile syringes.

The vibrational spectra of the materials were acquired using a Nicolet 5DXB Fourier Transform Infrared (FTIR) spectrometer (Nicolet Instruments, Madison, WI, USA). The samples were prepared with 150 mg of previously dried potassium bromide (KBr) and 1.5 mg of each sample, corresponding to 1.0% (w/w). Spectra were recorded in the range of 4000 to 400 cm^−1^ with a resolution of 4 cm^−1^, using a minimum of 64 scans per sample.

We also used Scanning Electron Microscopy (SEM) to study the morphology of the particles. For this, 110 µg/mL of GOX-materials were deposited onto a clean circular glass coverslip and allowed to dry under ambient conditions. The samples were then sputter-coated (SCD 050 - Balzers) with a thin layer of gold (~ 5 nm) to improve conductivity. SEM images were acquired using a Leo Stereoscan S440 operating under high vacuum and secondary electron mode (Everhart-Thornley Detector). The accelerating voltage was set to 10 kV, and images were taken at various magnifications.

### Cell culture

BEAS-2B (bronchial non-tumoral cell line), and HepG2 (liver cancer cell line) cells were cultivated in DMEM/F-12 culture medium supplemented with 10% fetal bovine serum. The cell lines are commercially available and were purchased from Banco de Células do Rio de Janeiro (BCRJ - Duque de Caxias, Brazil). The LC-HK2 (non-small cell lung cancer) cell line was established in the laboratory of Dr. Gláucia Machado-Santelli. The cell line has been previously characterized and described in several published studies, including analyses of cytoskeletal and nuclear alterations, as well as comparative characterization studies of human lung carcinoma cells (DOIs: https://doi.org/10.1007/s004180100262; https://doi.org/10.1007/s004180100262; https://doi.org/10.1590/1414-431X2023e12897 ; https://doi.org/10.1016/0309-1651(91)90155-C ). All the cell lines were maintained at 37 °C, with high humidity and in a controlled atmosphere containing 5% CO_2_. The culture medium was changed every two or three days, and the cells were subcultured through enzymatic dissociation with 0.2% trypsin and 0.02% EDTA solution.

### Experimental design and controls

For all experiments, cells were seeded and allowed to adhere for 48 h before treatment. GOX, GOXD, and GOXP were administered for 24 h at the indicated concentrations. Each experimental condition was performed in triplicate wells and repeated in three independent experiments. Negative controls consisted of untreated cells maintained under identical culture conditions. Blank controls containing nanomaterials in culture medium without cells were included to evaluate potential optical interference during absorbance measurements and fluorescence acquisition. A summary of the experimental design, including cell lines, treatment conditions, exposure times, and analyzed endpoints, is provided in Table 1 to facilitate clarity and comparability across assays.

**Table 1:** Summary of the experimental design for cell-based assays

Assay	Cell lines	Initial cell density	GOX, GOXD, GOXP concentrations	Exposure time	Replicates	Endpoint
Cell viability	BEAS-2B, LC-HK2, HepG2	BEAS-2B: 10 × 10^3^ LC-HK2: 10 × 10^3^ HepG2: 1 × 10^3^	0.032–160 µg/mL	24 h	*n* = 3 wells × 3 experiments	% viability, IC_50_
Cytoskeleton immunofluorescence	BEAS-2B, LC-HK2, HepG2	180 × 10^3^	110 µg/mL	24 h	100 cells / condition	Cell morphology and cytoplasmic area
Lamin B immunofluorescence	BEAS-2B, LC-HK2, HepG2	180 × 10^3^	110 µg/mL	24 h	100 cells / condition	Nuclear morphology and area

### GOX treatments

Stock solutions of GOX, GOXD, and GOXP were prepared at a concentration of 1 mg/mL in Phosphate-Buffered Saline without calcium and magnesium (PBS + A). Before each experiment, the materials were subjected to ultrasonic bath sonication for 30 min to promote homogeneous dispersion and minimize agglomeration. Working concentrations were freshly prepared by diluting the sonicated stock solutions in complete culture medium immediately before cell treatment.

The graphene-oxide concentrations used were 0.032, 0.32, 3.84, 38.4, 76.8, 128, and 160 µg/mL for the cell viability experiments. These concentrations were chosen because they correspond to 0.02, 0.2, 2.4, 24, 48, 80, and 100 µg/cm^2^ in dishes with a diameter of 3.2 cm.

For the immunofluorescence experiments, a concentration of 110 µg/mL was selected. This concentration was selected based on the viability results, as it represented a biologically active dose capable of inducing significant cellular responses. Although this concentration exceeded the IC_50_ in some cell lines, it allowed the assessment of cytoskeletal and nuclear alterations associated with cytotoxic effects while maintaining sufficient adherent cells for quantitative morphological analysis.

### Sulforhodamine-B assay for cell viability studies

The Sulforhodamine-B assay was performed to analyze cell viability in BEAS-2B, LC-HK2, and HepG2 cells exposed to GOX-derived materials at concentrations of 0.032, 0.32, 3.84, 38.4, 76.8, 128 and 160 µg/mL. For this, HepG2 cells were plated in a 96-well plate at a density of 5 × 10^3^ per well, and BEAS-2B and LC-HK2 cells at a density of 1 × 10^4^ per well. We utilized different cell densities to maintain the absorbance of the experimental control at 1, as recommended by the literature [34]. Cells were treated at the concentrations mentioned above for 24 h and fixed with 10% trichloroacetic acid (TCA) for 1 h at 4°C. Afterwards, we washed 3x with distilled water and added 0.4% Sulforhodamine-B for 30 min at 37 °C followed by washing 3x with 1% acetic acid. The crystals were solubilized with 10 mM Tris Base and the absorbance was read in a spectrophotometer at a wavelength of 570 nm. Absorbance values were normalized to untreated control cells, which were set as 100% viability. Background correction was performed using blank wells containing medium and nanomaterials without cells.

Half-maximal inhibitory concentration (IC_50_) values were estimated by linear interpolation using the equation:

$IC_{{50}} ~ = ~C_{1} + \left[ {\left( {50 - Y_{1} } \right)/\left( {Y_{2} - Y_{1} } \right)} \right] \times \left( {C_{2} - C_{1} } \right)$, where C_1_ and C_2_ are the concentrations immediately below and above 50% cell viability, respectively, and Y_1_ and Y_2_ are the corresponding % cell viability values.

### Cytoskeleton immunofluorescence

These experiments were performed to evaluate morphological changes in the cytoskeleton organization when cells were exposed to GOX-based materials. The cells were fixed with 3.7% formaldehyde, the plasma membrane was permeabilized with 0.5% Triton X-100 for 20 min. After that, cells were incubated overnight with a primary monoclonal anti-α tubulin antibody inside a moist chamber. After three washes with PBS + A, the cells were incubated for 2 h with the fluorochrome-conjugated secondary anti-mouse antibody. Subsequently, F-actin was stained with Alexa 555-phalloidin for 60 min and the nuclei were stained with 1 µg/mL of 4’,6-diamidino-2phenylindole (DAPI). Finally, the cytological preparations were mounted on the microscopic slide with Vectashield (Vector) and observed under a fluorescence microscope (Biotek, Winooski, VT, USA). For each condition, at least ten random microscopic fields were acquired per experiment under identical exposure settings. Image acquisition parameters were kept constant across treated and control groups to ensure comparability.

Cell area quantification was performed in FIJI (ImageJ, NIH) using fluorescence images stained for microtubules, actin filaments, and nuclei. Image channels were separated and converted to grayscale to improve contrast. The outer boundary of each cell was manually delineated using the freehand selection tool, following the visible continuous cytoskeletal fluorescence signal. The nuclear area was manually outlined based on DAPI fluorescence. Only isolated cells with clearly distinguishable borders were included in the analysis. One hundred randomly selected cells per condition were analyzed. Statistical analyses were performed using one-way ANOVA in GraphPad Prism.

### Nuclear lamina labelling by anti-lamin B immunofluorescence

To better evaluate the interaction of GOX-based materials with cell nuclei, fluorescent labeling of the nuclear lamina protein, lamin B, was performed through an immunofluorescence experiment. For this, BEAS-2B, HepG2, and LC-HK2 cells were plated in 35 mm dishes with 18 × 18 mm glass coverslips for 24 h at a density of 18 × 10^4^ per dish. Then, GOX, GOXD, and GOXP treatments were performed at 110 µg/mL for 24 h. After, the cells were washed three times with PBS + A, fixed with 3.7% formaldehyde for 30 min, permeabilized with 0.5% Triton X-100 for 20 min and incubated with primary anti-lamin B antibody (obtained from rabbits, purchased from Abcam (UK) in a humid chamber overnight. Three washes were then performed, and the cells were incubated for 2 h with a secondary anti-rabbit antibody conjugated with fluorochrome-CY5. After that, the nuclei were stained with 1 µg/mL of DAPI for 30 min following the instructions by the manufacturer. Finally, the preparations were mounted with Vectashield (Vector) and observed under a Lionheart fluorescence microscope (Biotek, Winooski, VT, USA).

## Results and discussion

**Fig. 2:**
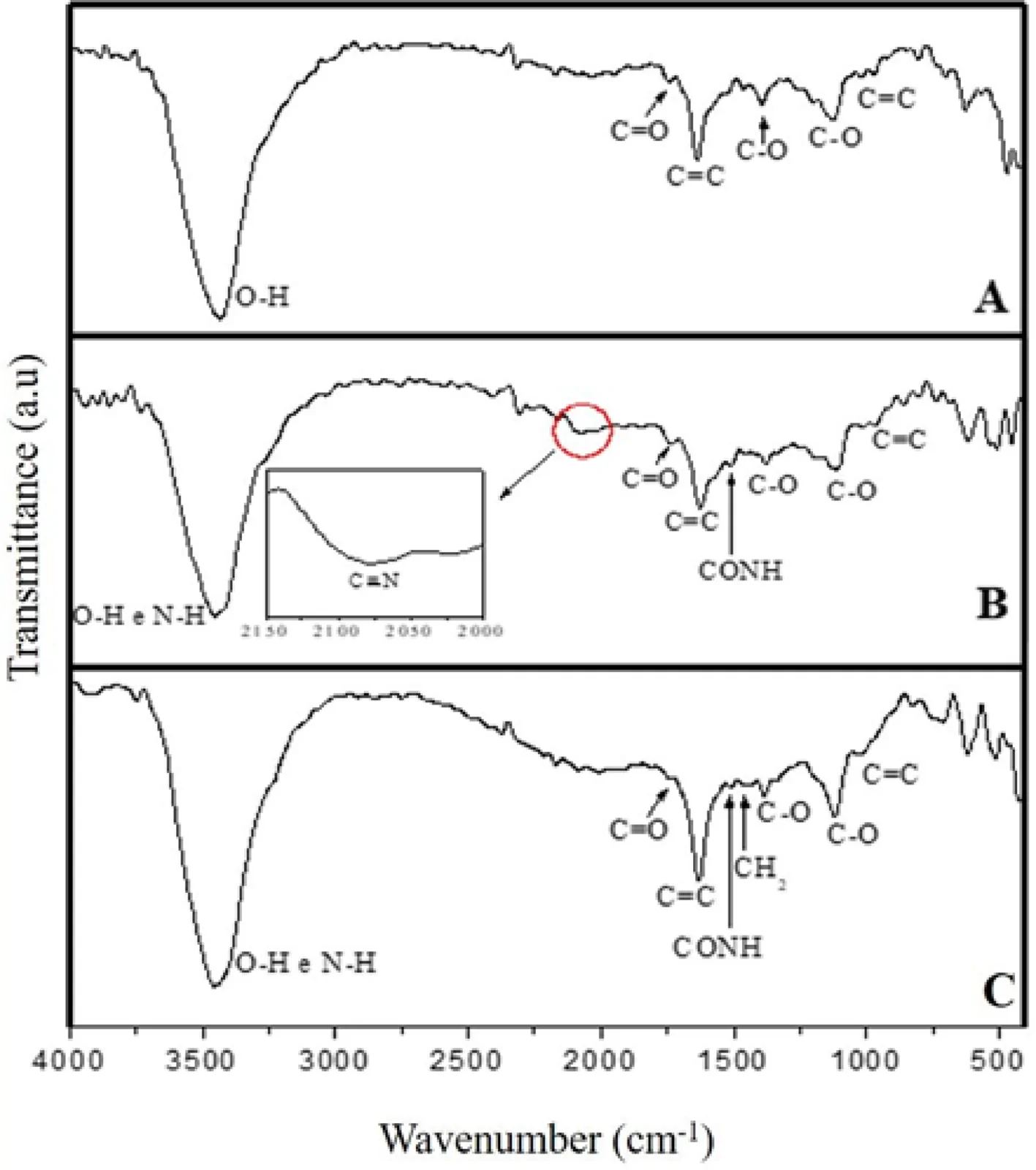
Vibrational spectra (FT-IR) for **A** GOX, **B** GOXD and **C** GOXP

- Particle characterization:

The FT-IR spectra of GOX (A), GOXD (B), and GOXP (C) are presented in Fig. 2. The spectrum of GOX shows characteristic bands of graphene oxide, including a broad O–H stretching band (3713–3066 cm^−1^), C =O stretching at 1736 cm^−1^, C =C stretching at 1630 cm^−1^, and C–O vibrations at 1383 and 1115 cm^−1^, confirming the presence of oxygen-containing functional groups on the basal plane and edges.

Following dendrimer functionalization, both GOXD and GOXP spectra exhibit features consistent with amide bond formation. In particular, the appearance of bands attributed to CONH vibrations (1512 cm^−1^ for GOXD and 1633 cm^−1^ for GOXP), together with characteristic CH_2_ stretching bands, confirms successful covalent attachment of dendrimers to GOX. The broad absorptions between 3728 and 3058 cm^−1^ further reflect contributions from hydroxyl and amine groups introduced by the dendrimer structure.

The presence of amide-related vibrations provides direct evidence of chemical grafting between dendrimer terminal amine groups and GOX carboxyl moieties, and not simple physical adsorption. This modification is expected to alter surface charge distribution, hydration behavior, and interfacial interactions, thereby influencing colloidal stability and subsequent nanoparticle-cell interactions.

A comprehensive structural characterization of GOXD and GOXP, including X-ray Photoelectron Spectroscopy (XPS), Thermogravimetric Analyzer (TGA), and elemental analysis confirming covalent functionalization, has been previously reported [7, 8]. The present FT-IR data are consistent with those findings and further validate the chemical modification of GOX in the formulations used for the biological assays.

Nanoparticle size determination is a crucial element in the evaluation of nanoparticles for cellular internalization, sensing, cellular acceptance, and toxicity [35].

The hydrodynamic diameter and zeta potential of the formulations were determined by dynamic light scattering (DLS). It is important to note that DLS assumes spherical particles, which represents a limitation when applied to graphene oxide-based materials due to their two-dimensional morphology. Therefore, the reported hydrodynamic diameters correspond to the size of dispersed aggregates, and not individual nanosheets.

The size distribution curves are presented in Supplementary Fig. S1. GOX exhibited a bimodal distribution with peaks at approximately 200 and 751 nm. GOXD showed a predominant peak centered around 532 nm. GOXP displayed two main peaks at approximately 100 nm, attributed to PAMAM, and 532 nm, indicating the presence of smaller components and larger assemblies. The Z-average hydrodynamic diameters were 1261.7 ± 186.9 nm for GOX, 927.5 ± 17 nm for GOXD, and 1863 ± 356.6 nm for GOXP (Table 2), reflecting the heterogeneous and polydisperse nature of the suspensions. Zeta potential values were − 34.5 mV (GOX), − 24.1 mV (GOXD), and − 7.3 mV (GOXP). GOX exhibited comparatively higher colloidal stability, whereas GOXD showed moderate stability.

The progressive reduction in negative surface charge from GOX to GOXP suggests partial neutralization of GOX carboxyl groups by positively charged dendrimer amines. This reduction in electrostatic repulsion may promote interparticle interactions, explaining the higher aggregation tendency observed for GOXP. Surface charge has been shown to be a primary determinant of nanoparticle-cell interactions, governing cellular uptake and cytotoxicity even in the presence of a protein corona, with less negatively charged or near-neutral particles exhibiting distinct biological behavior compared with strongly anionic counterparts [36, 37, 38]. Additionally, studies of nanoparticle surface chemistry indicate that changes in surface charge influence the composition and stability of the protein corona and consequent cell interactions, underscoring the importance of considering electrostatic properties when interpreting biological responses to modified nanomaterials [39].

**Fig. 3:**
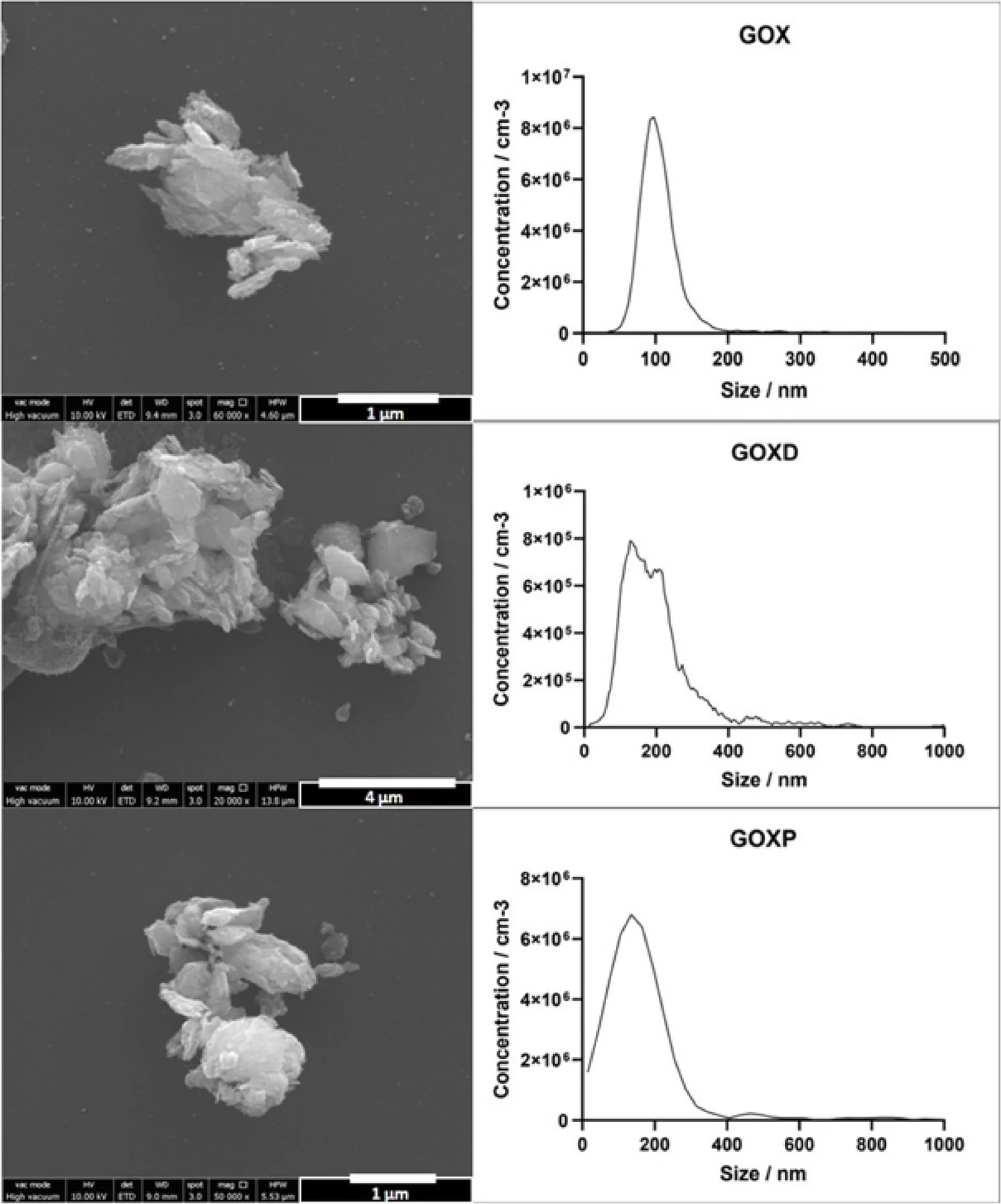
Particle size characterization of graphene oxide (GOX) and functionalized derivatives.** A**: Graphene oxide particles observed by scanning electron microscopy (SEM).** B**: Size distribution of graphene oxide particles obtained by nanoparticle tracking analysis (NTA).** C**: Graphene oxide functionalized with DAB-Am-16 observed by SEM.** D**: Size distribution of GOX + DAB-Am-16 particles obtained by NTA. **E**: Graphene oxide functionalized with PAMAM observed by SEM. **F**: Size distribution of GOX+PAMAM particles obtained by NTA

To further characterize particle size distribution in suspension, individual particle tracking analysis (NTA) was performed (Fig. 3). GOX particles have an average diameter of 107 ± 35 nm, whereas the GOXD particles tend to aggregate into clusters which broadens the peak of the size distribution, with an average diameter of 206 ± 123 nm. The GOXP particles show a similar aggregation pattern with an average diameter of 166 ± 118 nm. In contrast to GOX and GOXD, GOXP has a substantial concentration of nanoparticles of the order of 10 nm, which overlaps with the size of PAMAM.

DLS has known limitations in the characterization of non-spherical and polydisperse graphene-related materials, often leading to overestimation of hydrodynamic diameters due to the strong contribution of larger aggregates to the scattering signal (). In contrast, nanoparticle tracking analysis (NTA) provides number-based size distributions and enables improved resolution of coexisting particle populations in heterogeneous suspensions [40]. Accordingly, the discrepancy between the micrometer-scale Z-average values obtained by DLS and the smaller mean diameters measured by NTA reflects the heterogeneous and aggregation-prone nature of the suspensions, particularly following surface functionalization.

Importantly, the presence of smaller nanoparticle populations detected by NTA indicates that, despite aggregate formation, a fraction of nanoscale components remains dispersed in suspension. It has been shown that heterogeneous size distributions result in distinct modes of cellular interaction, since different endocytic pathways can be size-dependent [41, 42, 43]. Nanoparticles smaller than approximately 200 nm are more susceptible to clathrin- or caveolae-mediated internalization, and larger aggregates are more susceptible to be internalized through micropinocytosis or phagocytic processes, or predominantly interact with the cell membrane surface without efficient internalization [44]. The sedimentation of larger particle aggregates under static in vitro conditions can further increase cell surface exposure relative to the nominal concentration in the solution, leading to differential cellular responses [45, 46]. This size-dependent interaction profile highlights the importance of considering heterogeneous size distributions when interpreting nanoparticle-cell interactions and subsequent biological outcomes.

We also acquired images of the particles under the SEM (Fig. 3). This technique offers advantages such as the ability to generate images with a wide range of magnifications (20 to 30,000×), combined with high-resolution images (down to 1 nm) and depth of field [47]. We observed that the materials aggregated and formed clusters, but when isolated, they measured about 1 μm. This is similar to the work by Ibrahim et al. [48], which showed through SEM microphotographs that the formulated GO/PEG/Bru-FA NCs had a tetragonal and agglomerated appearance. The observation of micrometer-scale aggregates under SEM corroborates the aggregation trends detected by DLS and NTA. These aggregates can intensify sedimentation under in vitro conditions, increasing the effective exposure of nanoparticles on the cell surface and modulating particle-cell interactions, as reported in studies highlighting how sedimentation can dominate transport and internalization in adherent cultures. Larger and heavier nanoparticles tend to deposit more rapidly on cell monolayers, thus increasing the local dose received by cells relative to the nominal concentration in the solution. This phenomenon has been shown to influence internalization and biological responses in vitro, emphasizing the importance of considering aggregation and sedimentation in the interpretation of cell interaction data [49].

Together, these complementary techniques demonstrate that dendrimer functionalization modulates surface chemistry and colloidal behavior, resulting in heterogeneous, aggregation-prone suspensions with altered charge profiles. These parameters are critical determinants of nanoparticle-cell interactions and provide a mechanistic framework for interpreting the cellular responses described in the following sections.

Finally, it is important to consider that nanoparticle size and surface characteristics are dynamic parameters that may change upon dispersion in biological media. Upon exposure to complete cell culture medium, nanoparticles are subjected to physiological ionic strength and protein-rich conditions, which can promote protein corona formation and enhance interparticle interactions. These factors may lead to secondary aggregation or clustering over time, resulting in larger structures at the cell interface. Additionally, sedimentation under static in vitro conditions can increase local particle accumulation on the cell monolayer, contributing to the visualization of clusters on a micrometer scale. Therefore, the particle dimensions observed during cell assays reflect their dynamic behavior in biological media, and not their initial dispersion state [50, 51, 52].

**Table 2:** Zeta potential, Hydrodynamic diameter (Z-average Size), and polydispersity index (PdI) of GOX, GOXD, and GOXP formulations determined by dynamic light scattering (DLS). The hydrodynamic diameter represents the apparent size of the particles in suspension, which may reflect aggregate formation due to the two-dimensional morphology of graphene oxide-based materials. Measurements were performed in MiliQ water

Particles	Zeta potential	Size (d.nm)	PdI
GOX	− 34.5	1261.7 ± 186.9	0.5
GOXD	− 24.1	927.5 ± 17	0.6
GOXP	− 7.3	1863 ± 356.6	0.9

- Cell viability:

In vitro cytotoxicity assays are among the most important tools in toxicology applications and they are necessary to understand the impact of nanoparticles [53, 54]. We used the Sulforhodamine-B assay to evaluate cytotoxic effects, as this reagent measures cell density based on the measurement of cellular protein content [34]. The 50% inhibitory concentration (IC_50_) was determined by linear interpolation of dose-response data, representing the concentration of a substance required to inhibit 50% of a specific biological function [55].

**Fig. 4:**
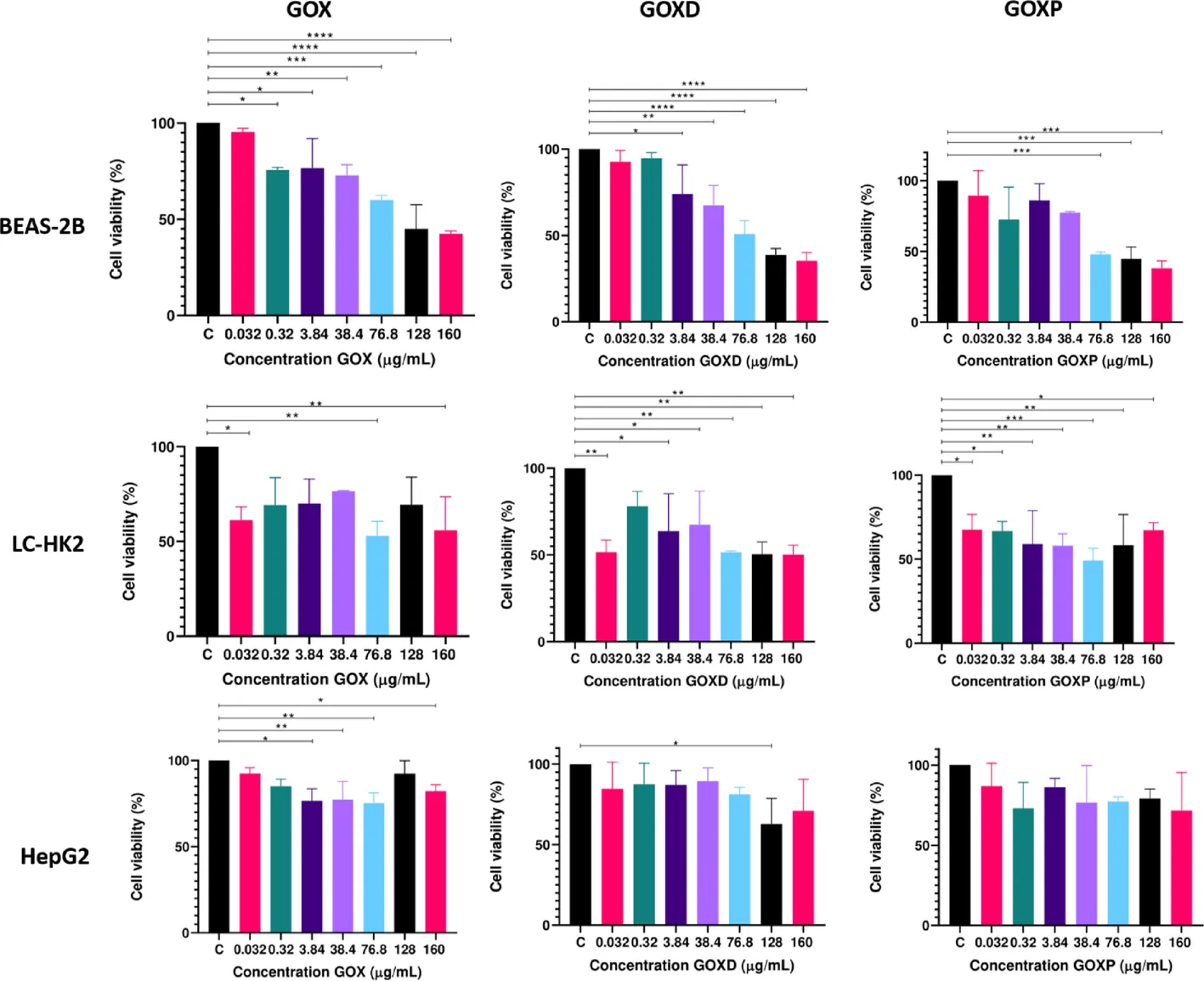
Cell viability of BEAS-2B, LC-HK2 and HepG2 evaluated by Sulforhodamine-B assay against exposure to different concentrations of GOX, GOXD and GOXP for 24 h. The bars represent the mean of three independent experiments and the standard deviation was calculated. Statistical significance was calculated by a one-way ANOVA test (*p < 0.05, **p < 0.005, ***p < 0.0005, ****p < 0.0001)

Our results showed that for BEAS-2B cells, GOX exhibited an IC_50_ of 111 µg/mL, GOXD of 80 µg/mL, and GOXP of 74 µg/mL (Supplementary Fig. 2); for LC-HK2 cells, GOX and GOXD exceeded 160 µg/mL (the highest concentration tested), whereas GOXP was 76 µg/mL (Supplementary Fig. 3); and for HepG2 cells, all three nanomaterials showed IC_50_ values above the tested concentration range (Supplementary Fig. 4). Overall, cell viability tended to decrease with increasing concentrations, especially at higher doses (Fig. 4).

These results are consistent with the broader literature demonstrating that graphene oxide derivatives can exhibit low cytotoxicity in non-tumoral cells and cell-type-dependent responses, and that GOX induces no or low cytotoxicity [56, 57, 58]. For instance, carboxyl- and folate-functionalized graphene oxide nanosheets were reported to be comparatively non-toxic to non-tumoral cells even at higher concentrations, while also providing targeted tumor accumulation in vivo, highlighting their biocompatibility and potential as theranostic agents [59]. Similarly, graphene quantum dots (GQDs), including functionalized derivatives, showed minimal effects on human neural stem cells’ viability, proliferation, metabolic activity, and differentiation, indicating robust biocompatibility of well-engineered graphene materials [60]. In three-dimensional glioblastoma neurospheres, carboxylated and unfunctionalized GQDs modulated tumor malignancy by increasing membrane fluidity and inhibiting cell-to-cell interactions, demonstrating that surface functionalization can influence cellular responses and cytotoxicity profiles [61]. Finally, graphene-based nanoparticles conjugated with anticancer agents (GOX-Rg3-DOX) exhibited significant cellular association and maintain minimal toxicity in Huh7 cells, suggesting that surface modification and conjugation strategies can mitigate oxidative stress and enhance biocompatibility, even in cancer cell models [62]. Oxidative stress has been suggested as a general toxicity mechanism for graphene derivatives [12, 63]. Kalman et al. [64] on the other hand demonstrated that graphene materials did not alter lysosomal function and did not induce ROS formation in hepatocytes. Liao et al. [65] described the toxic effects of GOX on A549 cells by regulating gene transcription, immune response, cell growth, and apoptosis, through the pathway analysis of SMARCA4, TGF-β1, and TP53. Castagnola et al. [66] described good biocompatibility of GOX and few-layer graphene (FLG), which did not impact the integrity and functionality of the barrier. However, direct comparison between studies is limited due to potential differences in graphene oxide layer number, lateral size distribution, and surface functionalization. Such physicochemical variations are known to significantly influence biological responses. Therefore, although our findings indicate relatively lower cytotoxicity under the tested conditions, further structural characterization would be necessary to enable direct comparison with previously reported materials.

The difference observed in cell viability results between the cell lines can be explained by a combination of factors. First, cellular responses to nanomaterials are highly dependent on the cell type, due to differences in membrane composition, metabolic activity, and sensitivity to oxidative stress [67, 68, 69]. For example, BEAS-2B and LC-HK2 cells (bronchial epithelial cells and non-small cell lung cancer cells, respectively) may respond differently compared to HepG2 (hepatoblastoma) cells due to their distinct physiological roles and antioxidant defenses [70, 71]. This may explain why the IC_50_ was only reached in BEAS-2B cells for GOX and GOXP, while HepG2 did not reach the IC_50_ at any of the concentrations tested. Second, the chemical modifications between GOX, GOXD, and GOXP could also account for the differences, particularly regarding surface charge, functional groups, or interaction with biological molecules. GOXD, for instance, showed higher cell viability at low concentrations in BEAS-2B cells, which may be due to increased cellular tolerance of the material. In contrast, GOXP seems to be more cytotoxic at lower concentrations in BEAS-2B cells, possibly due to the nature of the final surface functionalization. Therefore, the observed differences likely result from a combination of both variables: the intrinsic properties of each cell line and the specific characteristics of each material.

**Fig. 5:**
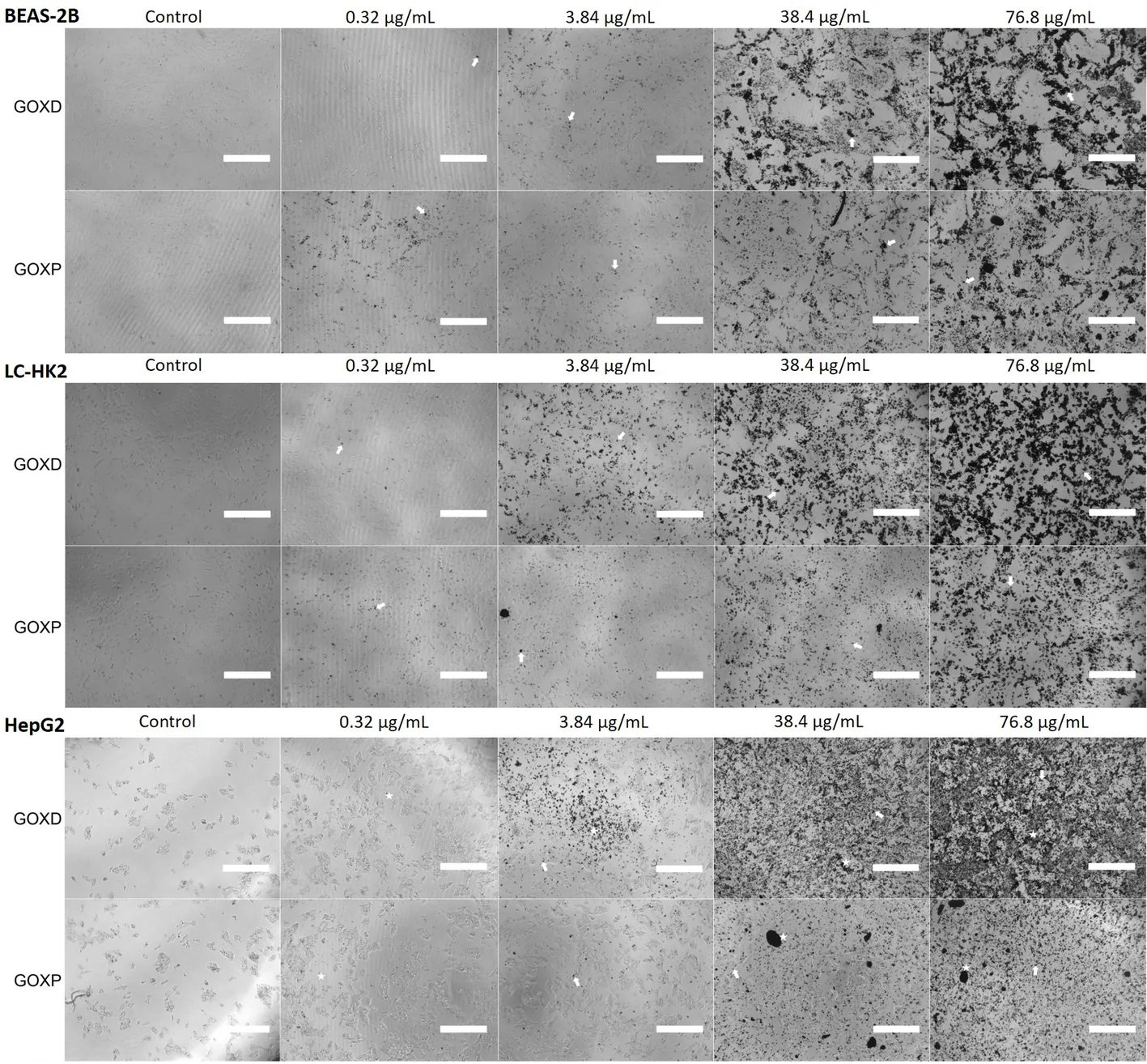
Images of BEAS-2B, LC-HK2 and HepG2 cells with different concentrations of GOXD and GOXP obtained with an optical microscope. The arrows highlight the presence of the graphene-oxide material on the cell surface. Scale bars: 400 μm

The increase in cell viability observed at higher concentrations in LC-HK2 and HepG2 cells may reflect a hormetic-like response, characterized by a biphasic dose-response pattern when low to moderate stress levels stimulate adaptive cellular mechanisms. Hormesis has been described in cancer biology and toxicology as a phenomenon with sublethal stress that enhances metabolic activity or activates cytoprotective pathways, without necessarily reflecting increased proliferation. In hepatocyte-derived models such as HepG2, this effect may be associated with activation of antioxidant and stress-response pathways. The concept of hormesis in cancer-related contexts has been discussed in the literature [72. 73, 74]. Nevertheless, additional assays evaluating oxidative stress markers would be required to confirm this mechanism in future studies (Figure 5).

- Cytoskeleton immunofluorescence:

We performed cytoskeletal immunofluorescence experiments to study the interaction between graphene oxide-based materials and cellular components after cell exposure.

Although the concentration used for immunofluorescence experiments (110 µg/mL) approaches or exceeds the IC_50_ in certain cell line-material combinations, it was selected to allow reliable detection of cytoskeletal alterations while preserving sufficient viable cells for structural analysis. It is important to emphasize that concentrations used in in vitro nanomaterial studies do not directly translate to systemic in vivo exposure levels. Cellular responses depend strongly on exposure conditions, local particle accumulation, and assay configuration. In vitro assays are employed to elucidate mechanistic interactions between nanomaterials and cellular components, and concentrations near or slightly above IC_50_ are frequently used to reveal sublethal structural alterations that may not be detectable at lower doses. Moreover, extrapolation of in vitro nanoparticle concentrations to in vivo settings requires careful interpretation due to differences in biodistribution and effective tissue exposure [75, 76].

**Fig. 6:**
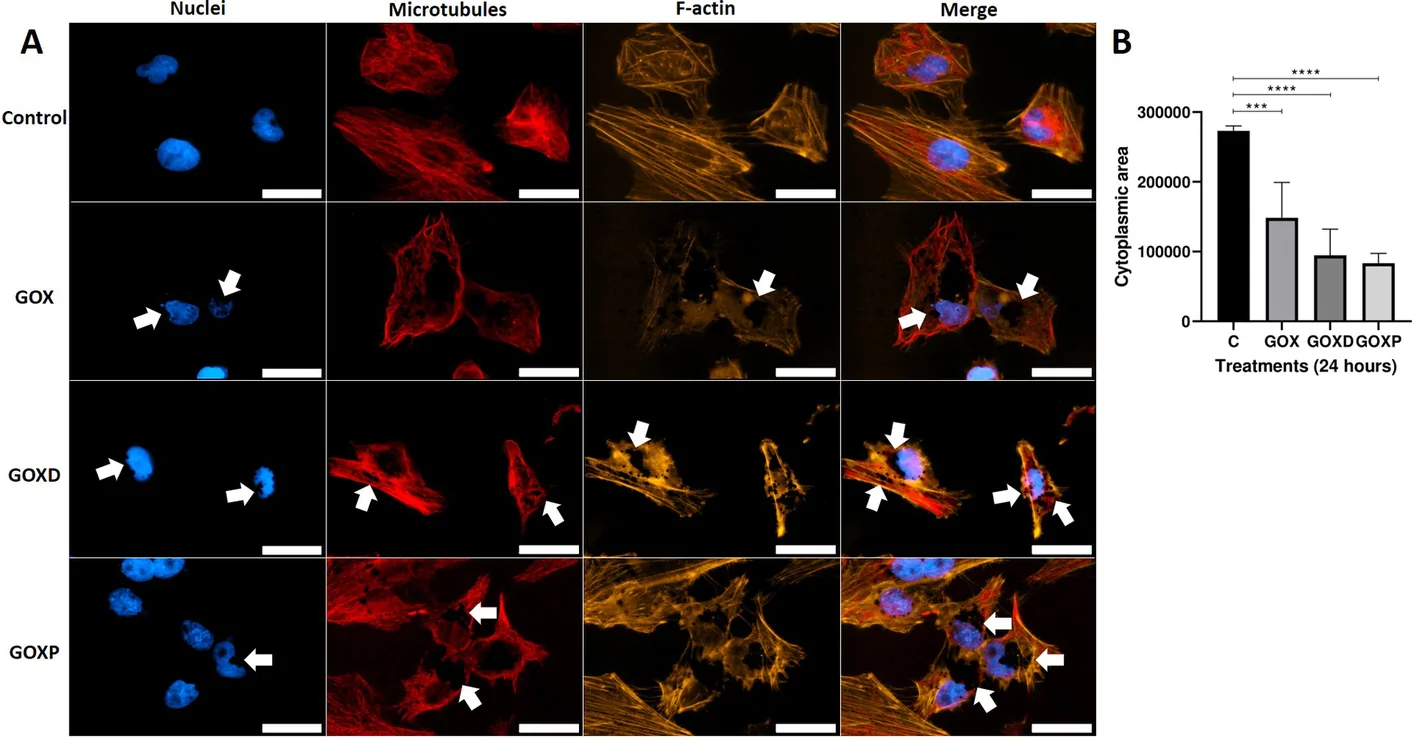
**A** Immunofluorescence of LC-HK2 cells showing microtubules stained with anti-α tubulin monoclonal antibody and secondary antibody with CY5. Actin microfilaments stained with phalloidin-Alexa 555 and nuclei stained with DAPI. The cells were exposed to 110 µg/mL of GOX materials for 24 h. The images were obtained using fluorescent microscopy. Arrows: the presence of material associated with the cytoskeleton and nuclei. Scale bars: 20 μm.** B** Measurement of cytoplasmic area (in pixels) performed using FIJI software (ImageJ, National Institutes of Health). Statistical significance was calculated by a one-way ANOVA test (***p < 0.001, ****p < 0.0001)

**Fig. 7:**
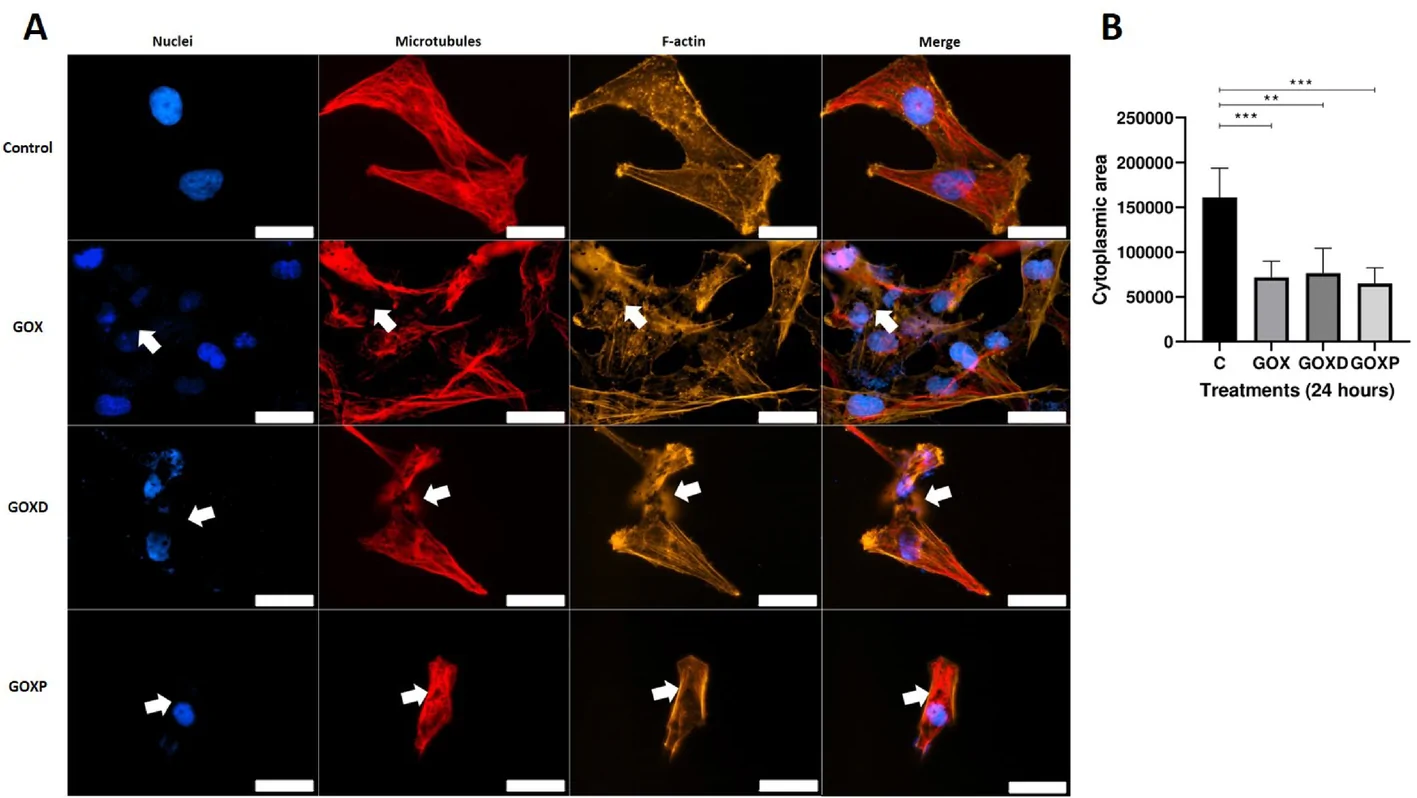
**A** Immunofluorescence of BEAS-2B cells showing microtubules stained with anti-α tubulin monoclonal antibody and secondary antibody with CY5. Microfilaments stained with phalloidin-Alexa 555 and nuclei stained with DAPI. The cells were exposed to 110 µg/mL of GOX materials for 24 h. The images were obtained using fluorescent microscopy. Arrows: the presence of material associated with the cytoskeleton and nuclei. Scale bars: 20 μm.** B** Measurement of cytoplasmic area (in pixels) performed using FIJI software (ImageJ, National Institutes of Health). Statistical significance was calculated by a one-way ANOVA test (**p < 0.005, ***p < 0.001)

**Fig. 8:**
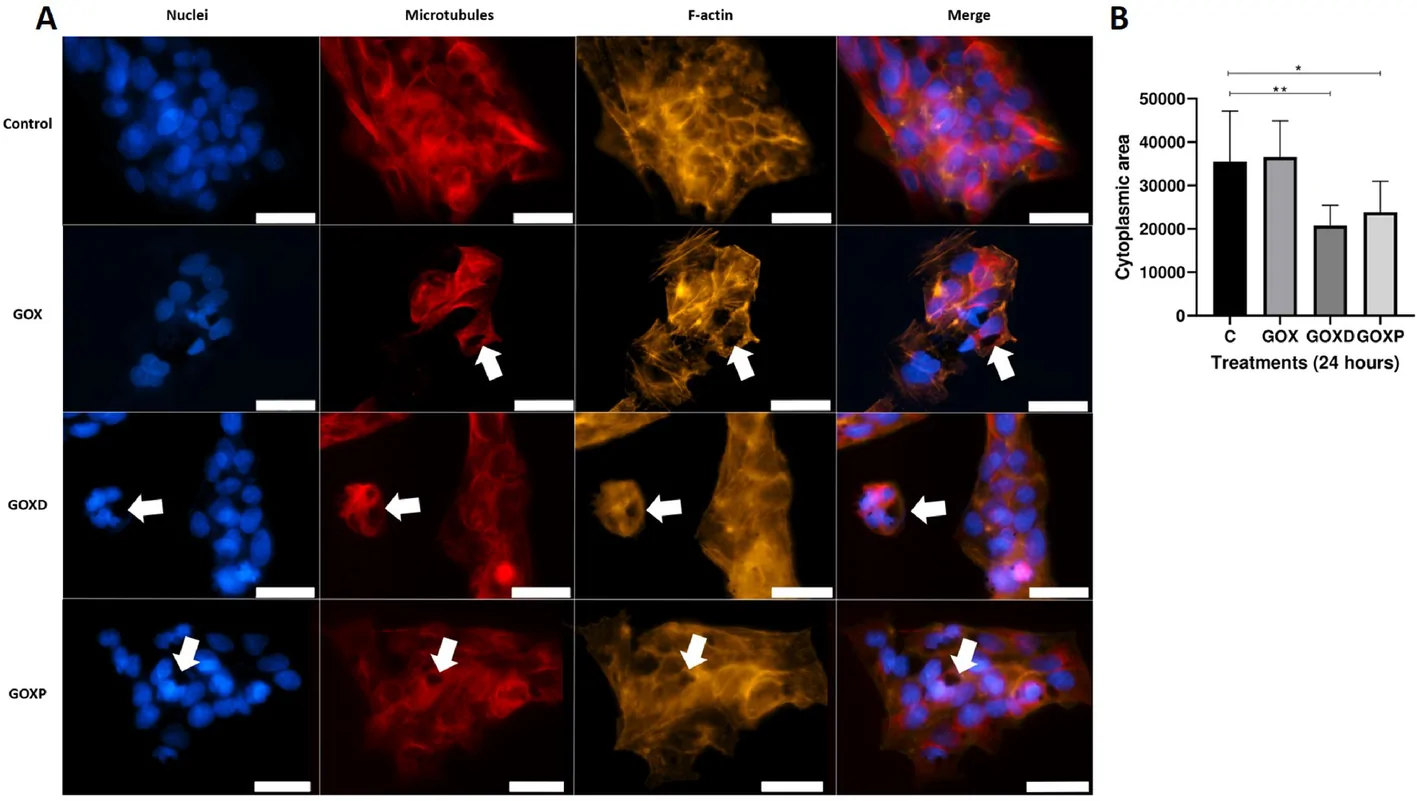
**A** Immunofluorescence of HepG2 cells showing microtubules stained with anti-α tubulin monoclonal antibody and secondary antibody with CY5. Microfilaments stained with phalloidin-Alexa 555 and nuclei stained with DAPI. The cells were exposed to 110 µg/mL of GOX materials for 24 h. The images were obtained using fluorescent microscopy. Arrows: the presence of material associated with the cytoskeleton and nuclei. Scale bars: 20 μm.** B** Measurement of cytoplasmic area (in pixels) performed using FIJI software (ImageJ, National Institutes of Health). Statistical significance was calculated by a one-way ANOVA test (*p < 0.05**p < 0.005)

Based on image analysis, a reduction in cytoplasmatic area was observed after 24 h of exposure to the nanomaterials in all cell lines (Figs. 6, 7 and 8). This may be linked to alterations in cytoskeletal components, which play a central role in maintaining cell shape, structure, and mechanical integrity [77, 78]. Microtubules, a key component of the cytoskeleton, are composed of polymerized α- and β-tubulin dimers. Their dynamic polymerization is involved in various cellular processes, including mitosis, cytokinesis, and vesicular transport [79]. These cytoskeletal alterations may have functional consequences for the cells, potentially affecting processes such as mitosis, cytokinesis, vesicular transport, and migratory capacity, even in the absence of evident cytotoxicity. Such sublethal structural changes highlight that cell viability alone may not fully reflect preserved cellular functionality [80, 81].

Bera et al. [82] demonstrated the effect of GOX on the tubulin structure, showing that GOX disrupted the structural integrity of the protein, consequently retarding tubulin polymerization. Additionally, GOX inhibited microtubule formation by interfering with the polymerization of tubulin heterodimers both in vitro and in vivo, resulting in growth arrest at the S phase and ROS-induced apoptosis of HCT116 colorectal carcinoma cells. Ghorbani et al. [83] described that GOX nanosheets affected the biological properties of MCF-7 and MCF10a cells by the disruption of the cytoskeleton induced conformational changes in actin filaments, altering themicrofilament network and decreased cell migration and stiffness in both cell lines, without causing cell death. Chen et al. [84] demonstrated that graphene oxide disrupts the actin cytoskeleton specifically in cancer cells, thereby inhibiting their migratory capacity, while non-cancer cells remain largely unaffected. They suggested that the interaction between thin GOX sheets and the plasma membrane of cancer cells serves as a trigger for cytoskeletal disorganization.

**Fig. 9:**
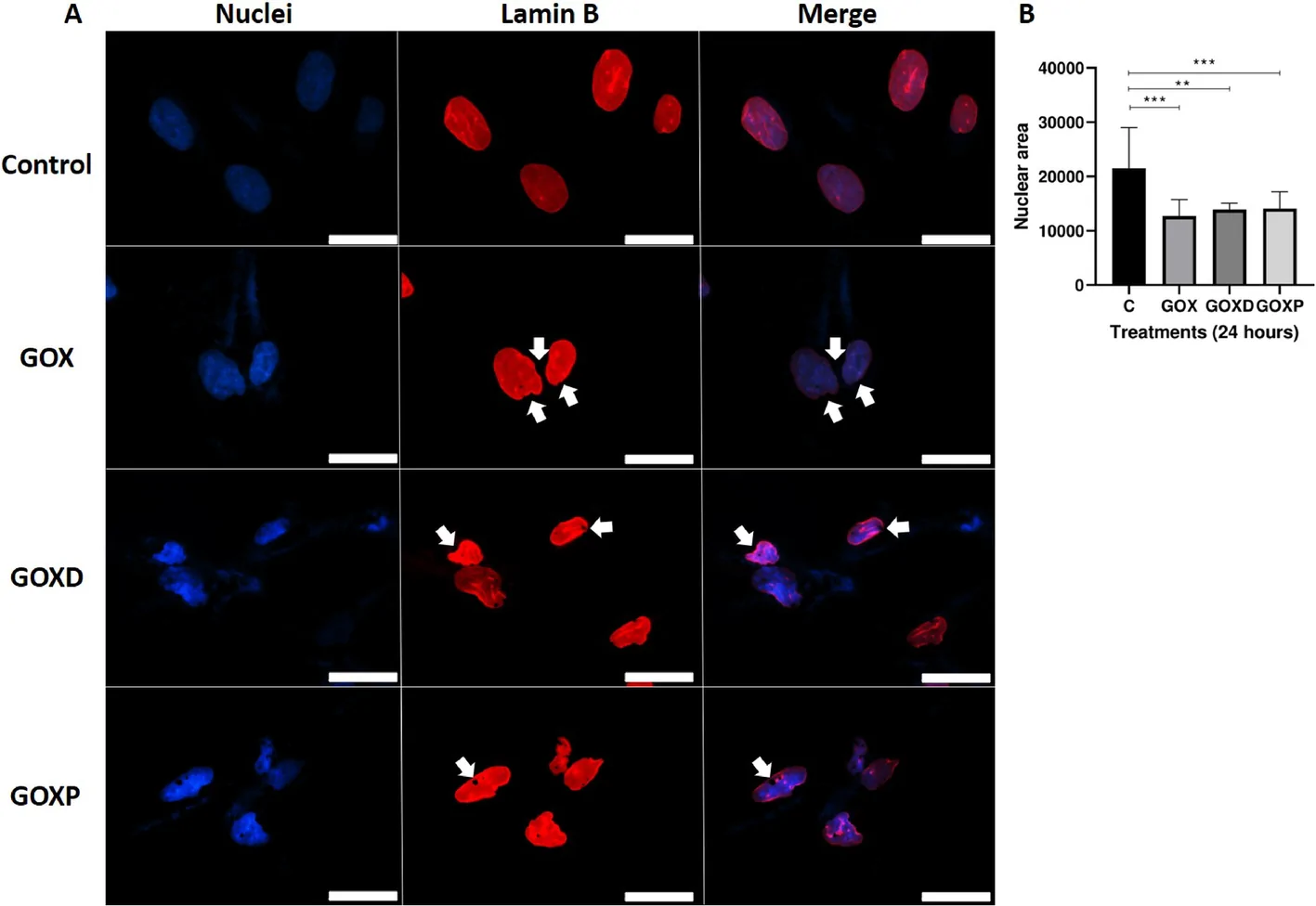
Immunofluorescence images of BEAS-2B cells. BEAS-2B cells were exposed to 110 µg/mL of the materials for 24 h. Control cells received only culture medium. **A**: in red: immunofluorescence highlighting the nuclear lamina with anti-lamin B antibody and secondary antibody anti-rabbit with CY5; in blue: nuclei stained with DAPI. Scale bars: 30 μm. Arrows highlight the interaction between the particles and the cells. **B**: measurement of nuclear area (pixels) performed using FIJI software (ImageJ, National Institutes of Health). Statistical significance was calculated by a one-way ANOVA test (**p < 0.005, ***p < 0.0005)

**Fig. 10:**
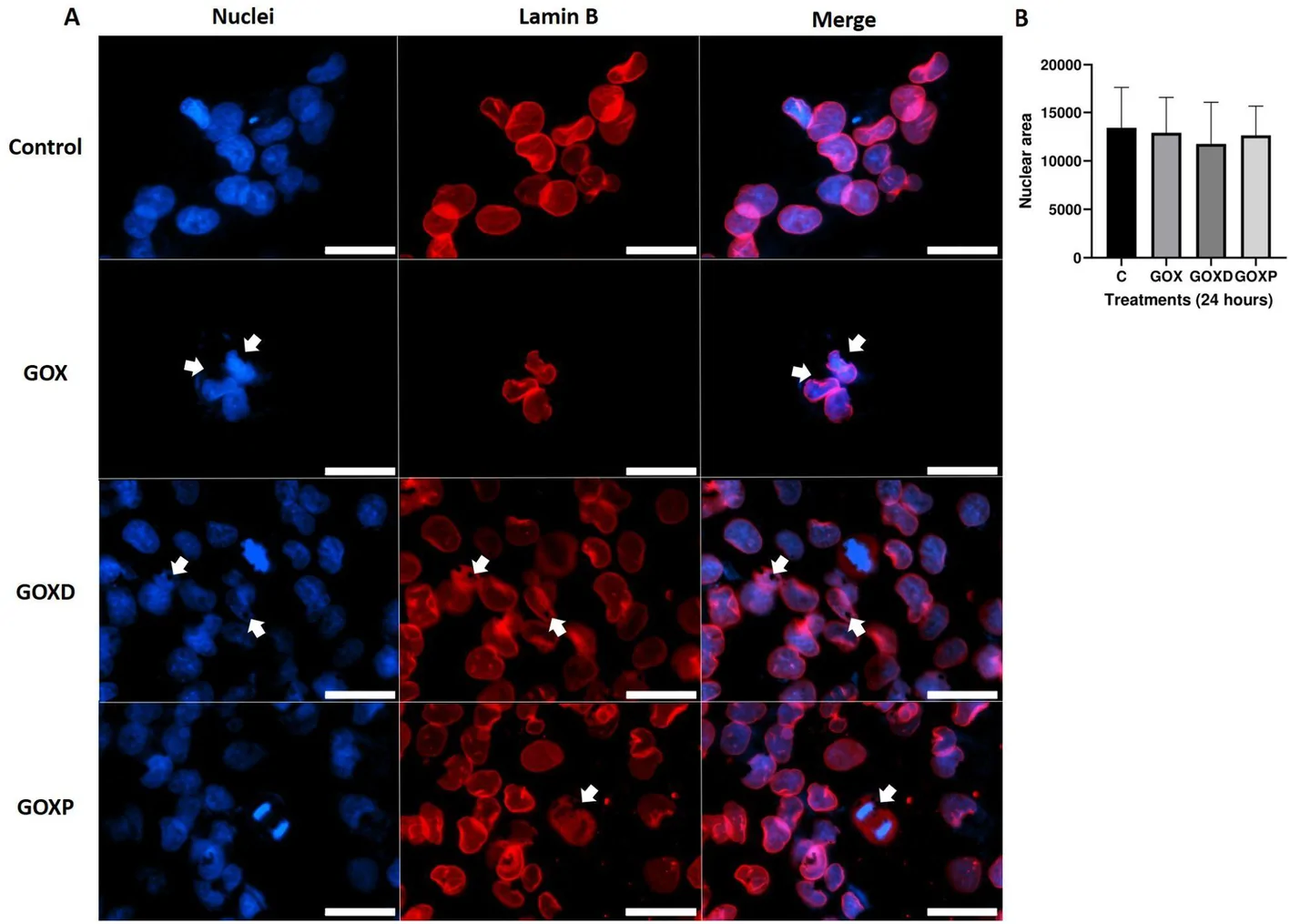
**A** LC-HK2 cells exposed to 110 µg/mL of GOX, GOXD and GOXP for 24 h. In red: immunofluorescence highlighting the nuclear lamina with anti-lamin B antibody and secondary antibody anti-rabbit with CY5; in blue: nuclei stained with DAPI. Scale bars: 30 μm. Arrows highlight regions of morphological change caused by the interaction of the particles and the cells. **B** Measurement of nuclear area (pixels) performed using FIJI software ImageJ, National Institutes of Health). Statistical significance was calculated by a one-way ANOVA test

**Fig. 11:**
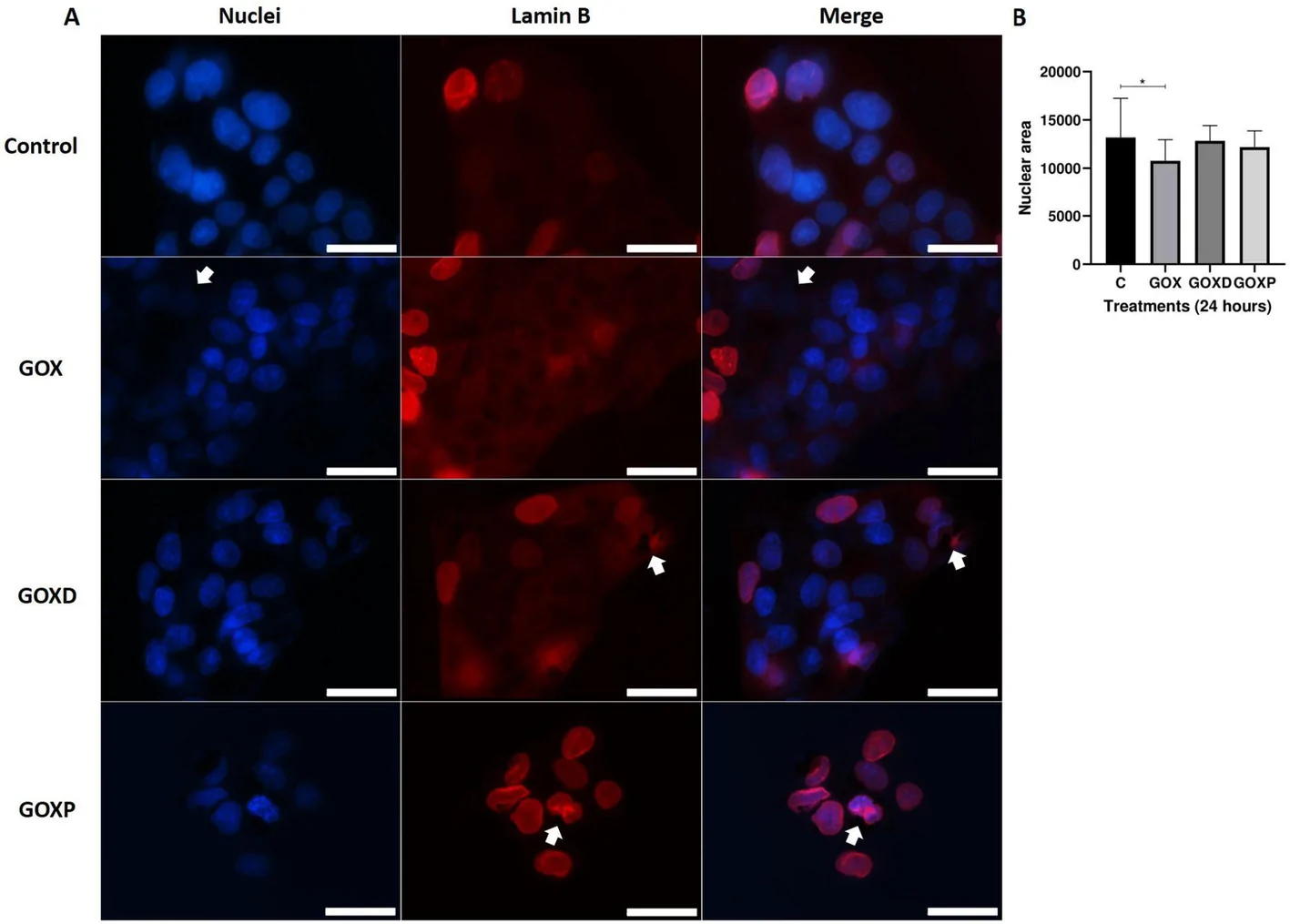
HepG2 cells exposed to 110 µg/mL of GOX for 24 h. In red: immunofluorescence highlighting the nuclear lamina with anti-lamin B antibody and secondary antibody anti-rabbit with CY5; in blue: nucleus stained with DAPI. Scale bars: 30 μm. Arrows highlight regions of morphological change caused by the interaction of the particles and the cells. B) Measurement of nuclear area (pixels) performed using FIJI software ImageJ, National Institutes of Health). Statistical significance was calculated by a one-way ANOVA test (*p < 0.05)

**Fig. 12:**
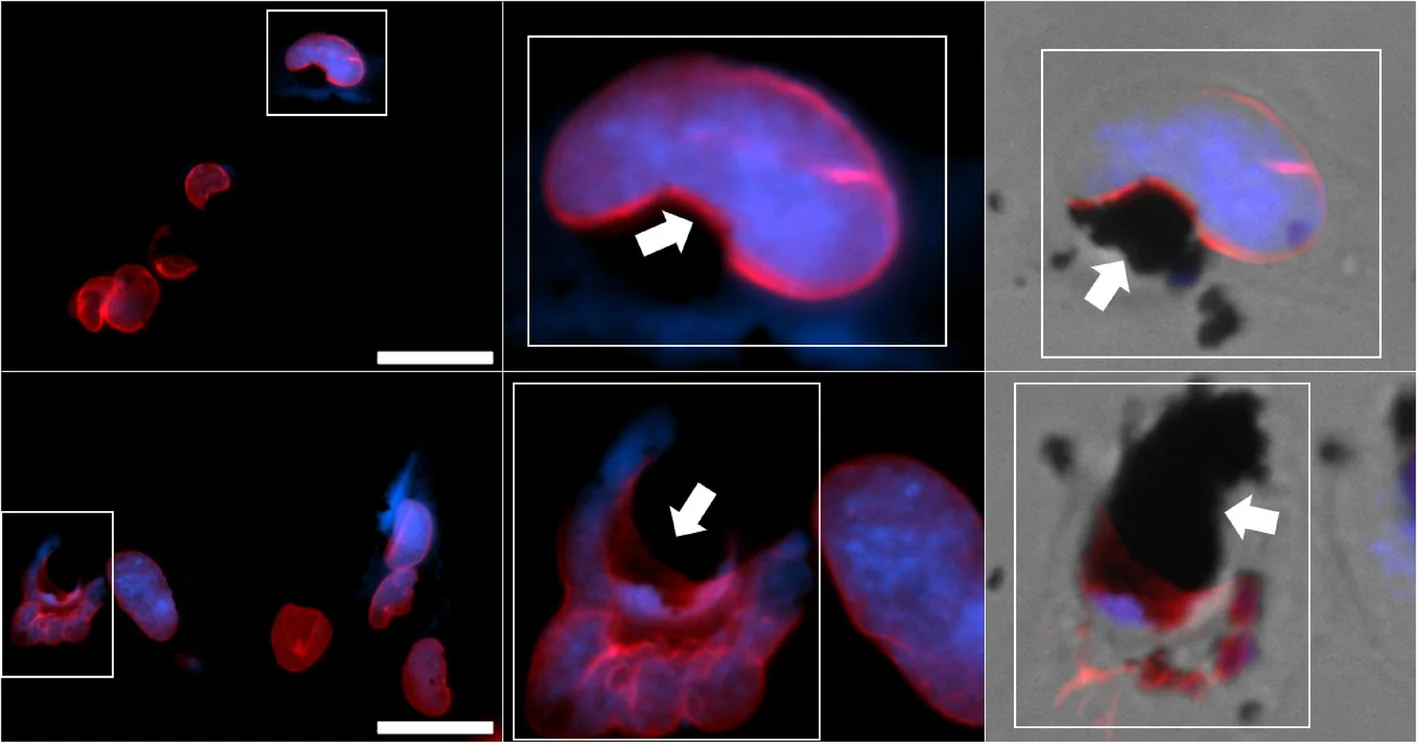
Further details of LC-HK2 cells exposed to 110 µg/mL of GOX for 24 h. In red: immunofluorescence highlighting the nuclear lamina with anti-lamin B antibody and secondary antibody anti-rabbit with CY5; in blue: nucleus stained with DAPI. Scale bars: 30 μm. Arrows highlight regions of morphological change caused by the interaction of the particles and the cells

- Lamin B immunofluorescence:

Given the observed spatial interaction between the graphene-based materials and the cell nuclei, we investigated the potential effects of these nanomaterials on the nuclear envelope. For this purpose, we conducted fluorescent labeling to assess nuclear morphology. Furthermore, morphometric analysis demonstrated a significant decrease in nuclear area in BEAS-2B cells following exposure to graphene oxide-based materials, relative to untreated control (Fig. 9). In contrast, no statistically significant differences were observed in LC-HK2 cells under the same conditions (Fig. 10). For HepG2 cells, a significant reduction in nuclear area was detected only in those treated with GOX (Fig. 11). Despite the evident accumulation of particles around the nuclear periphery and the apparent mechanical compression exerted on the envelope, no signs of membrane rupture or structural discontinuity were detected (Fig. 12). These findings suggest that these materials may induce nuclear compaction and morphological alterations as a result of mechanically induced nuclear remodeling, without causing membrane rupture.

Similar to the cell viability results, the differences observed in nuclear area measurements may be attributed to the intrinsic properties of each cell line and the specific physicochemical characteristics of the graphene-based materials. Additionally, HepG2 cells tend to grow in clumps or domes, which may hinder the direct interaction between the nanomaterial and individual cells, potentially reducing its intracellular effects [85]. BEAS-2B, as a non-tumorigenic epithelial cell line, may be more vulnerable to nanomaterial-induced damage compared to LC-HK2 cells, a tumor-derived cell line that may exhibit enhanced resistance due to altered cellular metabolism and stress response pathways [84].

The reduction in nuclear area observed in our study is consistent with morphological alterations induced by other nanoparticles. Xu et al. [86] reported that graphene quantum dots can accumulate within the nucleus, leading to changes in nuclear area and shape, as well as DNA damage. This also resembles the findings by Jin et al. [87], who showed that GOX was internalized by A549 cells and observed, through transmission electron microscopy, to be associated with the nucleus and cytoplasm, without causing significant damage to the morphology of the organelles.

## Conclusions

Although there is an impairment in cell viability, most of the tested concentrations for the graphene oxide-based materials were above the IC_50_. Additionally, strong interactions between the cells and the materials were observed by microscopy analysis. Morphological studies revealed that exposure to these materials was associated with alterations in cytoskeletal organization, including a reduction in both cytoplasmic and nuclear area. These cytoskeletal changes may impair essential cellular functions, such as division and motility, indicating that cell survival does not necessarily reflect fully preserved functionality. Taken together, these findings indicate acceptable cytotoxicity of the materials within specific concentration ranges and support their potential for further investigation in biomedical applications. Nevertheless, this study is limited to in vitro analyses, and further studies - including more extensive functional assays and in vivo evaluations - are required to fully assess the safety, biodistribution, and therapeutic potential of these materials.

## Supplementary Information

Below is the link to the electronic supplementary material.

Supplementary Material 1

## Data Availability

Data is available upon request from the authors.
