# Supplementary material for: Evaluating cellular responses of lung and liver cells to graphene oxide functionalized poly(propylene)imine and polyamidoamine dendrimers: insights for biomedical applications
Source: Discov Nano. 2026 Sep 11;21(1):435. doi: 10.1186/s11671-026-04636-0 (PMC13569771; doi:10.1186/s11671-026-04636-0)
Supplement: Supplementary file 1 [file 11671_2026_4636_MOESM1_ESM.docx]

**Figure S1.** Intensity-based hydrodynamic size distribution profiles of GOX, GOXD, and GOXP obtained by dynamic light scattering (DLS). Samples were sonicated for 30 min and then dispersed in MilliQ water (10 µg/mL) for the measurements.

**Figure S2.** Dose-response curves of BEAS-2B cells exposed to GOX, GOXD and GOXP. Cell viability (%) is shown as the mean of three independent experiments, and the standard deviation is shown as SD. IC₅₀ values were calculated by linear interpolation following the equation mentioned in Methods.

**Figure S3.** Dose-response curves of LC-HK2 cells exposed to GOX, GOXD and GOXP. Cell viability (%) is shown as the mean of three independent experiments, and the standard deviation is shown as SD. IC₅₀ values were calculated by linear interpolation following the equation mentioned in Methods.

**Figure S4.** Dose-response curves of HepG2 cells exposed to GOX, GOXD and GOXP. Cell viability (%) is shown as the mean of three independent experiments, and the standard deviation is shown as SD. IC₅₀ values were calculated by linear interpolation following the equation mentioned in Methods.

**Figure S5**. Thermogram of: GOX (A), GOXD (B), and GOXP (C) (7,8).

**Table S1.** Atomic percentage of GOX, GOXD and GOXP elements constituents (7,8)**.**

| **Elements** | **Atomic %** | | |
| --- | --- | --- | --- |
|  | GOX | GOXD | GOXP |
| Carbon (C 1s) | 89.6 | 86.9 | 84.3 |
| Oxygen (O 1s) | 10.0 | 8.2 | 11.7 |
| Nitrogen (N 1s) | - | 5.1 | 4.0 |

**Figure 6S**. XPS of GOX (A), GOXD (B), and GOXP(C) showing full range, high resolution C 1s, high resolution O 1s, and high resolution N 1s (7,8).
